# A qualitative approach to guide choices for designing a diary study

**DOI:** 10.1186/s12874-018-0579-6

**Published:** 2018-11-16

**Authors:** Karin A. M. Janssens, Elisabeth H. Bos, Judith G. M. Rosmalen, Marieke C. Wichers, Harriëtte Riese

**Affiliations:** 1 Interdisciplinary Center for Psychopathology and Emotion regulation, Department of psychiatry, University Medical Center Groningen, University of Groningen, Groningen, the Netherlands; 2 Sleep-Wake Center, Stichting Epilepsie Instellingen Nederland (SEIN), Zwolle, The Netherlands; 3 Developmental Psychology, University of Groningen, Groningen, the Netherlands

**Keywords:** Ecology momentary assessment, Experience sampling methods, Diary design, qualitative research

## Abstract

**Background:**

Electronic diaries are increasingly used in diverse disciplines to collect momentary data on experienced feelings, cognitions, behavior and social context in real life situations. Choices to be made for an effective and feasible design are however a challenge. Careful and detailed documentation of argumentation of choosing a particular design, as well as general guidelines on how to design such studies are largely lacking in scientific papers. This qualitative study provides a systematic overview of arguments for choosing a specific diary study design (e.g. time frame) in order to optimize future design decisions.

**Methods:**

During the first data assessment round, 47 researchers experienced in diary research from twelve different countries participated. They gave a description of and arguments for choosing their diary design (i.e., study duration, measurement frequency, random or fixed assessment, momentary or retrospective assessment, allowed delay to respond to the beep). During the second round, 38 participants (81%) rated the importance of the different themes identified during the first assessment round for the different diary design topics.

**Results:**

The rationales for diary design choices reported during the first round were mostly strongly related to the research question. The rationales were categorized into four overarching themes: nature of the variables, reliability, feasibility, and statistics. During the second round, all overarching themes were considered important for all diary design topics.

**Conclusions:**

We conclude that no golden standard for the optimal design of a diary study exists since the design depends heavily upon the research question of the study. The findings of the current study are helpful to explicate and guide the specific choices that have to be made when designing a diary study.

**Electronic supplementary material:**

The online version of this article (10.1186/s12874-018-0579-6) contains supplementary material, which is available to authorized users.

## Background

Diary studies in which participants are asked to repeatedly fill out questions about experienced feelings, cognitions, behaviors and their social context are increasingly being performed [1]. This is probably due to technological developments that ease performance of diary studies, such as the wide availability of Smartphones. Further, awareness is growing that the use of repeated assessment of (psychological) symptoms makes it possible to acquire valuable insights about (psychological) dynamics that cannot be obtained with the use of single-administered questionnaires [2]. The terms commonly used in diary research are experience sampling methods (ESM) and ecological momentary assessment (EMA). The terms ESM and EMA each stand for a wide variety of ambulatory assessment methods ranging from paper diaries and repeated telephone interviews to electronic data recording technologies and physiological recordings with sensors [3]. ESM/EMA aims to measure symptoms, affect and behavior in close relation to experience and context [3, 4].

When designing a diary study, several choices have to be made. First, the research question should be specified, since the research question has important consequences for the choices of a diary design. Second, a decision on the sampling design should be made, e.g. the duration of the diary study and the measurement frequency. Third, one has to decide on the number of items to include in the diary. Fourth, a decision has to be made on whether participants have to answer the questionnaires on predefined (i.e. fixed assessment) or at random time-points. Fifth, it should be decided whether the items are about the here-and-now (i.e. momentary assessment) or about a previous time period (i.e. retrospective assessment). Finally, one has to decide on the delay participants are allowed to respond to the prompt to fill out the questionnaire. This is the time allowed to complete the survey before it is counted as missing data.

So far, diary design issues have mostly been addressed in methodological papers and textbooks (e.g., [4–8]). Although these are very useful, they are typically based on extensive personal experience from researchers who work in a specific research field. A systematic overview of information on diary design from researchers from different research fields is lacking. Typically, specific details on argumentation of choosing a particular diary design are not described in scientific publications. In order to increase information on diary designs performed in different disciplines, Stone and Shiffman already plead for careful description of argumentations for choosing a particular diary design in scientific papers [8]. Nevertheless, recent research indicates that the rationale for these choices is often not reported [9]. To overcome this omission in the literature, the aim of the current study was to obtain insight into reasons behind diary design by performance of a qualitative study. As a first step, we wanted to identify key elements relevant to diary design shared by research fields. Therefore, experts on diary studies from different research fields were asked about the rationale behind their choices for particular diary design topics.

## Methods

### Study population

All members of the international Society of Ambulatory Assessment [10] were invited by e-mail to participate in the study. Additionally, we invited researchers experienced in diary research from our personal network. Finally, PubMed was searched using the search terms “diary studies” and “time-series analysis”, after which leading authors of relevant articles were approached. Since the optimal design for a diary study depends heavily upon the research question, we did not strive to reach consensus, as opposed to a typical Delphi study. However, we believed that a second assessment round was necessary to validate the themes that we identified during the first assessment round. Therefore, our study is a semi-Delphi study [11] in which answers reported by the researchers during a first assessment round were independently summarized and anonymously given back to all participating researchers for feedback during a second assessment round. The first and second assessment round consisted of online questionnaires. All data processing and feedback reports were done without disclosing the identity of the participating researchers.

### Procedure

The first assessment round ran between October 2015 and February 2016, the second assessment round ran between April 2016 and July 2016. During both assessment rounds participants were provided a link to a Google Docs questionnaire by e-mail. Participants could answer all questions online. During the first assessment round, only open text fields were used. During the second assessment round, questions were answered on either 10 point rating scales or entered in open text fields. The data processing of both assessment rounds is explained in more detail below.

### First assessment round

In the first round of this study, participants were asked to answer questions about themselves and the amount of experience they had with performing diary studies. Next, they were specifically asked about a diary study they had designed or co-designed. They were requested to report on the type of participants, study duration, the measurement frequency, the use of random or fixed assessments, the choice for momentary or retrospective assessment, and the delay participants were allowed to respond. In the current research, retrospective assessment means that questions covered the period between two prompts. In contrast to regular research in which retrospective recall is often much longer, this period was up to 24 h in the diary studies reported about in our research. We distinguished retrospective assessment from momentary assessment. During momentary assessment, questions covered the few minutes before the participants were filling out the questionnaires. Participating researchers were asked to provide these characteristics of their diary design and thereafter for their rationale behind these choices. Finally, we asked whether they would use the same diary design when he/ she should redo the study.

### Data processing of the first round

KJ performed the initial thematic analysis to group the reasons given for particular diary designs into different categories for each diary design topic. A second rater (HR) grouped all arguments into these categories, and added or skipped categories if needed. In case no consensus was reached about the grouping of the arguments, a third rater (EB) made the final decision. The categorized rationales were grouped in overarching themes during a consensus meeting in which all authors (i.e., KJ, EB, JR, MW and HR) participated. During this meeting additional questions on the overarching themes raise, namely questions about the number of items used in the diary, practical suggestions and observed hiatus in the literature. These questions were added to the second assessment round.

### Second assessment round

The overarching themes containing the categorized rationales for diary designs were given back to the participants during the second assessment round, in the form of a textual description accompanied by bar graphs depicting the frequency of the reported rationales. These, slightly adapted, bar graphs can be found in the result section of this article. Researchers were asked to rate the importance of the identified themes for diary studies in general for all the diary design topics (i.e. study duration, sampling frequency, random or fixed design, momentary or retrospective assessment, and delay allowed to respond to the beep). This was done on a rating scale ranging from 1 (not important at all) to 10 (extremely important).

### Data processing of the second round

For each diary design topic, the median importance rate and interquartile range (IQR) were computed for each theme. The open text fields were thematically analyzed in the same way as during the first assessment round, except that EB was the second rater, and HR the third rater for the questions about practical suggestions and reported hiatus in the literature.

## Results

### First assessment round

#### Participants

Forty-seven researchers participated in our study and provided us with information of 47 different diary studies. Two researchers reported about the same diary study during the first round, and one researcher reported about two studies during the second round. Answers about characteristics of the same study given by multiple participating researchers were only counted once. The researchers worked at 27 different institutes from 12 different countries. Details about the participating researchers are given in Table 1. Information about the studies they reported on are given in Table 2.

**Table 1:** Characteristics of participating researchers (*n* = 47)

Age (years)	39 (IQR 31,47)	
Female	23 (49%)	
Experience in conducting diary studies (years)	5 (IQR 2, 10)	
Number of diary studies (co)designed (number)	3 (IQR 2, 7)	
Countries	The Netherlands	26 (10 institutes)
	United States of America	7 (7 institutes)
	Germany	3 (2 institutes)
	Switserland	2 (2 institutes)
	Belgium	2 (1 institute)
	Israel	1
	Austria	1
	Australia	1
	United Kingdom	1
	Finland	1
	China	1
	Spain	1

Note: *IQR* Interquartile range

**Table 2:** Characteristics of studies reported about (*n* = 47)

Type of participants in diary studies	Only healthy participants	21 (45%)
	With somatic symptoms	8 (17%)
	With psychiatric symptoms	17 (36%)
	With somatic and psychiatric symptoms	1 (2%)
Momentary or retrospective	Momentary	14 (30%)
	Retrospective	12 (25%)
	Both	21 (45%)
Fixed or random assessment	Fixed	24 (52%)
	Random	11 (24%)
	Semi-random	10 (22%)
	Fixed and random	1 (2%)
Use study design again	Yes	27 (60%)
	No	17 (40%)
	Median (Interquartile range)	Mode (Range)
Study duration (days)	17 (7, 30)	14 (1, 270)
Measurement frequency (times/day)	5 (3, 10)	10 (1, 50)
Delay allowed to respond (minutes)	30 (15, 60)	60 (1.5, 1440)
Number of items in the dairy^a^	30 (19, 55)	5, 25, and 50 (5, 90)

^a^Assessed during the second round, based upon answers on 35 studies reported by 38 participating researchers

#### Study intensity

The median study duration of the studies reported on was 17 days (IQR 7, 30), the median sampling frequency was five times a day (IQR 3, 10) and the median number of items was 30 (IQR 19, 55). The longest study duration was 9 months with a sampling frequency of five times a day when the participant filled out 55 items. It should be noted that this latter study was a single-case study (*n* = 1) performed in a clinical setting. The highest sampling frequency was every 15 min for 1 day while the participants filled out seven items each time. The highest number of items participants had to fill out during a single assessment was 90 in a study with a sampling frequency of twice a day for a period of 30 days.

### Rationales for choices on different diary design topics

**Fig. 1:**
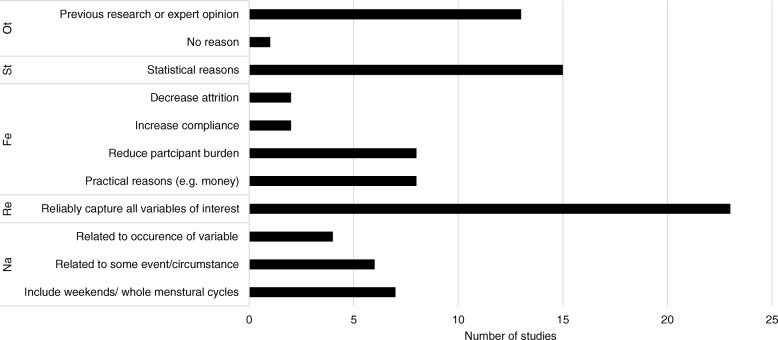
Overview of the reasons provided for the chosen study duration, grouped into themes: Ot = Other, St = Statistical reasons, Fe = Feasibility, Re = Reliability, Na = Nature of the variables

**Fig. 2:**
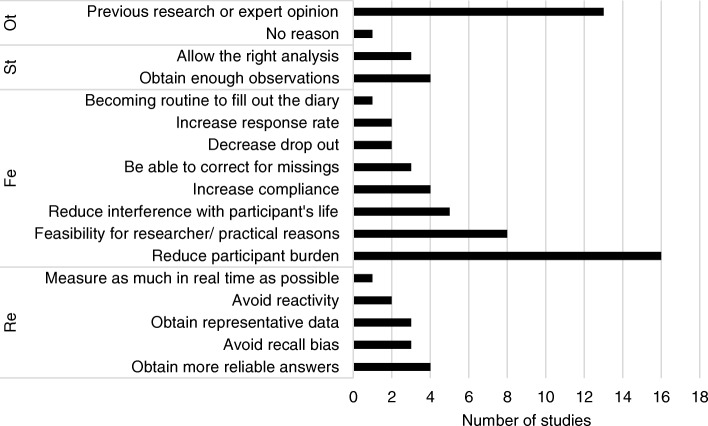
Overview of reasons reported for the chosen measurement frequency, grouped into themes: Ot = Other, St = Statistical reasons, Fe = Feasibility, Re = Reliability, Na = Nature of the variables

**Fig. 3:**
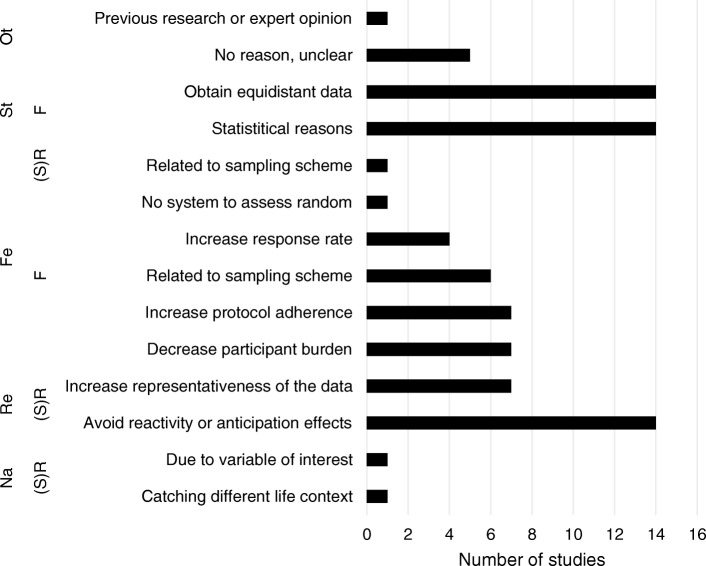
Overview of reasons provided for the choice of (semi)random and or fixed assessment, grouped into themes: Ot = Other, St = Statistical reasons, Fe = Feasibility, Re = Reliability, Na = Nature of the variables. F = fixed and (S)R = (semi-)random

**Fig. 4:**
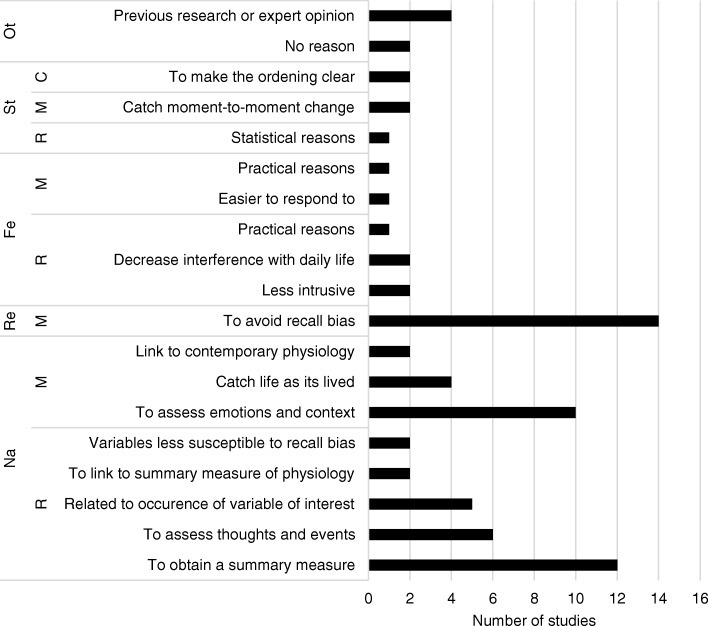
Overview of reasons provided for the choice of momentary or retrospective assessment, grouped into themes: Ot = Other, St = Statistical reasons, Fe = Feasibility, Re = Reliability, Na = Nature of the variables. M = Momentary, R = Retrospective and C = Combination of retrospective and momentary assessment

**Fig. 5:**
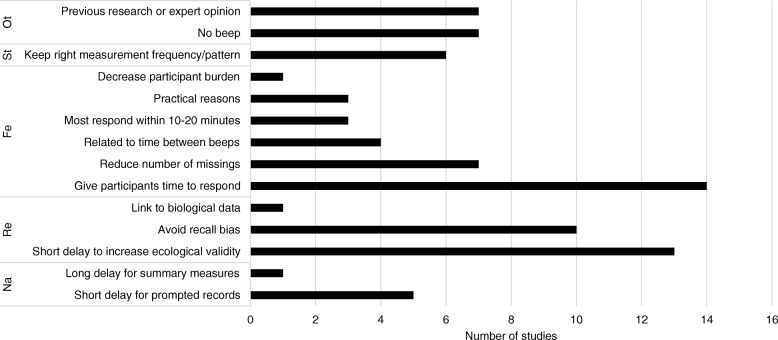
Overview of reasons provided for the chosen allowed delay to respond to the beep, grouped into themes: Ot = Other, St = Statistical reasons, Fe = Feasibility, Re = Reliability, Na = Nature of the variables

Rationales provided for the design choices will be discussed for each diary design topic, accompanied by bar graphs: i.e. the study duration (Fig. 1), measurement frequency (Fig. 2), random or fixed assessment (Fig. 3), momentary or retrospective assessment (Fig. 4), and allowed delay to respond to the beep (Fig. 5). Please note that some researchers did not provide a rationale for certain topics and that most researchers gave more than one rationale per topic. Therefore, the number of reasons does not add up to 47. A reason for a decision that was given for all design topics, was that the decision was based on previous research or expert opinion. Thirteen researchers did so for the decision on their study duration. They provided the following literature references: [12–17]. Thirtheen researchrs based their decision for the measurement frequency on previous research or expert opinion. The following references were given: [2, 3, 5, 12, 13, 16, 18, 19]. One researcher based the decision for momentary or retrospective assessment on previous literature. The following literature references were provided: [4, 20–25]. Four researchers based the decision for random or fixed assessment on expert opinion or previous literature. They provided the following literature references: [2, 24–27]. Finally, seven researchers based their decision for the allowed delay to respond to the beep on expert opinion or previous research. These literature references were provided: [12, 14, 17].

### Study duration

The median study duration was 17 days, with an IQR from 7 days till 30 days, and total range between 1 and 270 days. The most frequently reported reason for the choice of a study duration was to measure long enough to obtain reliable and representative data (23 times). Also statistical reasons, such as obtaining enough observations to perform specific statistical analyses, were often reported (15 times). Reducing participant burden was reported eight times for minimizing study duration. Six researchers wanted to make sure that their study duration captured both week and weekend days, or an entire menstrual cycle. For six researchers, the study duration was related to an event, such as 2 weeks before and 4 weeks after a studied intervention. Four researchers reported that their study had to include a longer period, since the variable of interest was a priori known to occur infrequently. Very practical reasons were reported as well, e.g. the limited battery life of the ambulatory sensor. The variety of reasons a researcher can have for a particular study duration is illustrated in the following response: *“We have chosen a measurement period of two weeks for several reasons. First, we wanted to have enough measurements to conduct reliable analyses within persons (….) we wanted at least 60 completed observations per person. We first decided that 5 measurements per day would be appropriate, and based on this, the minimum measurement period consists of 12 days. Eventually, we thought that 2 weekends in the measurement period would enable us to check weekend effects in a more reliable way, and we therefore set the measurement period at 14 days. In addition, our choice was also based on what we expected to be the maximum length that participants would be willing and able to fill out 5 measurements per day (….), and which length would be ‘representative’ for a person (….).”*

### Measurement frequency

The median measurement frequency was 5 times a day with an IQR range between 3 and 10 times a day, and total range between 1 and 50 times a day. Most researchers based their choice for a particular measurement frequency on the dynamics of the variable of interests (reported 18 times). Researchers interested in variables known to follow a circadian rhythm, like for example hormones or mood states, chose for relatively frequent measurements (e.g. 10 times a day). Researchers interested in variables expected to occur infrequently (e.g. panic attacks) or in summary measures (e.g. number of cups of coffee) chose infrequent measurements (i.e. once a day). Keeping acceptable participant burden was the second most often reported reason for a certain measurement frequency (reported 16 times). By keeping participant burden low, researchers wanted to diminish interference with participants’ normal daily lives (reported five times), improve compliance (reported four times), improve response rate (reported twice), and keep drop out low (reported twice). Nine researchers based their measurement frequency on the assessment on different parts of the day. Eight reported feasibility, not further specified, for their choice of the measurement frequency.

Five researchers chose for frequent measurements in order to increase representativeness of the data, and four to diminish recall bias and in this way increase reliability. Four researchers mentioned that frequent measurements were necessary to perform meaningful statistical analyses, and three that equidistant measurements were needed to meet the statistical assumptions, e.g. for vector autoregressive analyses. The ultimate choice for a particular study duration was mostly a compromise between these different aspects. This was nicely illustrated by one of the participating researchers: *“Because the study aimed to investigate a rapidly varying phenomenon, namely momentary affective state and reactivity, we wanted to optimize temporal resolution. We wanted as high measurement frequency (*i.e. *temporal resolution) as possible without compromising compliance. A frequency of 10 times per day has been shown to be feasible in previous studies (Csikszentmihalyi* et al*. 1987. J Nerv Ment Dis; 175:526–536, Shiffman* et al *2008. Annu Rev Clin Psychol; 4: 1–32).”*

### Random or fixed assessment

Although we asked whether researchers used fixed or random assessment, many researchers indicated that they used semi-random assessment instead. Semi-random assessment means that an assessment occurs at a random time point, however within a certain predefined time window, to ascertain that assessments are on average equally spread over a day. Twenty-four researchers used a fixed assessment, eleven used random assessment, ten a semi-random assessment, and one used a combination of fixed and random assessment design. Since we expect that random designs were in fact mostly semi-random in nature, arguments for using them will be discussed together.

(Semi-)Random assessment was mostly chosen to avoid reactivity and anticipation effects (reported 14 times) and to increase the representativeness of the data (reported seven times). For example, one researcher wrote: “*Random intervals presumably decrease the influence of the measurements on daily life activities of the participants, as the participants do not know the exact measurement time and cannot plan and change their activities based on that. This increases ecological validity. Semi-random intervals guarantee that measurement times are evenly distributed across the day (Shiffman et al 2008. Annu Rev Clin Psychol; 4: 1-32).”* The main reason for a fixed design was that such a design made it possible to obtain time-series data with equidistant time points required for many statistical techniques, such as vector autoregressive analyses (14 times). Other reasons for choosing for fixed designs were related to feasibility, e.g. to decrease participant burden (seven times), increase protocol adherence (seven times), and increase response rate (four times).

### Retrospective or momentary assessment

Most researchers (*n* = 21) used a combined momentary and retrospective assessment design, followed by 14 researchers that chose for only momentary assessments, and finally 12 researchers that chose for only retrospective assessment. A retrospective assessment design was often used in order to obtain a (reflective) summary measure (reported 12 times) and for assessing thoughts or events, since these are mostly count variables (reported six times). Momentary assessment was mostly used to assess current emotions or context (reported ten times) and to capture life as it is lived (reported four times). For example, one researcher wrote: *“Emotions were measured momentary as these are fleeting experiences and heavily influenced by recall bias. Daily events were asked retrospective (windows of approximately 90 minutes) as this is needed to capture the most important events that happened and that may have been rewarding or stressful”*. The most frequently reported reason to assess momentary was to diminish recall bias (reported 14 times). Reasons related to feasibility were reported for both assessment methods, e.g. retrospective assessment was considered to be less intrusive, and momentary assessment easier to respond to. With regard to statistics, two researchers used a combination of momentary and retrospective assessment to allow studying the temporal order of events: *“In addition, we chose for this design (affect/cognition momentarily and events retrospectively) because when assessing the relationships between events and affect/cognition the ordering would always be clear: events happened before the affect/cognition measurements. If both would have been asked retrospectively, it would have been less clear whether the affective states/cognitions came first or the events.”*

### Allowed delay to respond to the beep

The median amount of delay allowed to respond when respondents were prompted to fill out a questionnaire was 30 min, with an IQR of 15 min to 60 min, and total range between 1.5 min and 24 h. The most commonly reported reason for the delay allowed to respond was to give the respondents enough time to respond (reported 14 times). Thirteen researchers only allowed short delays in order to increase the ecological validity of the data (e.g., better representations of the activities the participant is currently involved in) and ten did so to diminish recall bias. Five researchers wanted to obtain momentary feelings and therefore did not allow participants much time to respond (that is < 20 min). Six researchers only allowed short delays to retain the measurement frequency needed to perform their analyses. The delay allowed to respond was also related to the measurement frequency. A researcher that opted for a long delay wrote: *“Because for time-series analysis it is important to have little missing values, we chose a relatively long delay. Because we only have three measurements a day and because many of our variables concern “the previous measurement interval”, we don’t see this as a big problem.”* A researcher who chose a short delay wrote*: “We chose to allow a relatively short length of delay to ensure the real-time assessment and to avoid recall bias. However, to increase compliance, some delay has to be allowed as the participants are living their normal lives and are not always able to reply immediately. 15 minutes delay has been used in previous studies (e.g. Jacobs et al 2011. Br J Clin Psychol; 50: 19-32). Results from previous paper&pencil –diary studies suggest that most of the participants answer within 10 to 20 minutes (Csikszentmihalyi et al 1987. J Nerv Ment Dis; 175:526-536) and that answers after a longer than 15 minutes delay are less reliable (Wichers et al 2007. Acta Psychiatr Scand; 115: 451-457).”*

### Using the study design again

Most researchers (i.e. *n* = 27 [60%]) reported that on hindsight they were satisfied with their designs. Of the researchers who were not satisfied, most would like to intensify their diary design, by using more frequent measurements (five researchers), extending the diary period (four researchers), using a combination of intensive diary and longitudinal designs (i.e. burst designs, two researchers), or adding some items (one researcher). Three researchers opted for a less intensive design: that is a lower measurement frequency (*n* = 2) or fewer items in the diary (*n* = 1). Further, two researchers would have included more participants and one would have personalized the diary items.

### Themes identified

After thematically analyzing the data of the first assessment round, four overarching themes were identified that covered the reasons mentioned for choices behind all diary design topics. The first theme was ‘the nature of the variables of interest’. This theme comprised reasons related to characteristics of the variable of interest, for example its fluctuation pattern or its occurrence rate. The second theme was ‘reliability’. Reliability referred to the reproducibility, representativeness, and/or consistency of the obtained assessments. The third theme was ‘feasibility’. This theme covered reasons related to practicability for both the participant and the researcher. The fourth theme was ‘statistics’. This theme contained reasons related to performance of statistical analyses. To get more insight into these categories, the reasons reported by the researchers are grouped per category in Figs. 1, 2, 3, 4, 5.

### Second assessment round

Thirty-eight participants (81% of the participants in the first assessment round) participated in the second assessment round. They rated the importance of the different overarching themes identified during the first assessment round for the choices of each diary design topic.

### Importance of different themes

The importance rates (scored on a scale from 0 = not important at all to 10 = extremely important) for the different overarching themes for each diary design topics are given in Table 3. The role of statistics for the choice of the time allowed to respond was considered least import (i.e. median 6, IQR: 4–8). The role of the nature of the variable of interest for the choice of momentary or retrospective was considered most important (i.e. median 9. IQR: 9–10). The nature of the variable(s) of interest (e.g., its occurrence or fluctuation rate) was found to be most important for making a decision about the measurement frequency and the choice for momentary or retrospective assessment. The nature of the variable(s) of interest and the reliability (e.g. obtaining representative data) were found to be most important for the choice of the study duration. The reliability and statistics (e.g. obtaining equidistant data) were most important for the choice of (semi)random or fixed assessment. The nature of the variable, reliability and feasibility were all found equally important for the choice of the delay allowed to respond.

**Table 3:** Importance rates of overarching themes for different diary design topics

	Nature of the variable	Reliability	Feasibility	Statistics
Study duration	9 (7, 9)	9 (7, 9)	8 (8, 9)	8 (7, 9)
Measurement frequency	9 (8, 10)	8 (7, 9)	8 (8, 9)	8 (6, 9)
Random or fixed assessment	7 (5, 8)	8 (7, 9)	7 (6, 9)	8 (6, 9)
Momentary or retrospective assessment	9 (9, 10)	8 (7, 9)	7 (5, 8)	7 (5, 8)
Delay allowed to respond	8 (7, 9)	8 (7, 9)	8 (7, 9)	6 (4, 8)

Note: Assessed on a scale ranging from 1 (not important) to 10 (extremely important), more details are given in the method section

Medians (Interquartile range) are given

### Practical suggestions

We will now discuss the practical suggestions to improve diary studies that were reported by more than one researcher. Suggestions that were reported by only one researcher are given in Additional file 1. Suggestions were provided by 34 researchers, four researchers did not report suggestions.

To increase the reliability of the obtained data, the following suggestions were given. Five researchers suggested to use language for the items and answering scales that suits participants (e.g. to use easy wording and ask about the here-and-now). Four researchers suggested to use previous studies or pilots to help designing a diary study. Two researchers suggested making the study relevant for participants as well, for example by providing them personalized feedback reports based on their diary data. Two researchers reported to use reliable items or modified traditional questionnaires. Two researchers suggested verifying the sampling times of the self-reported data with objective information obtained at the same moment (i.e. general available weather information) and telling participants that you will do so.

To increase the feasibility for participants, it was suggested to use short questionnaires (six times), sample not too frequently (five times), use electronic diaries or smartphones (four times), use fixed sampling designs (four times), provide incentives (four times), personalize the (fixed) sampling scheme to the participants’ preference (four times), and to allow a long delay to respond (twice).

Many suggestions were about involving participants when preparing, conducting, and evaluating the study. It was suggested to offer participants close support during the diary study (eight times), to think together with participants about how to prevent missing data (four times), give good briefing and instructions to your participants (four times), and to perform a pilot study to check on feasibility (twice). Other suggestions were to use fixed or equidistant assessment designs (four times) and to ensure enough assessments (four times) to increase statistical possibilities.

### Suggestions for future research

The nine suggestions for future research reported by more than one researcher were: theoretical guidance with regard to dynamics of phenomena of interest (ten times); theoretical guidance on what the advantages and disadvantages are of particular diary designs (eight times); information on how burdensome particular designs are (for particular target populations) (five times); information about power calculation (both number of participants and number of time-points) (four times); information on statistical strategies for diary data (thrice); information on optimal number of items (twice), information on how to obtain reliable data (twice): and information on whether the reliability of the data changes over time (twice). Gaps in the literature that were reported by only one researcher can be found in Additional file 2. Finally, five researchers indicated that they did not know the answer or did not respond, and three researchers reported that we know already a lot (although one of them also reported that an extensive/complete overview of all pros and cons of certain designs is lacking).

## Discussion

From the results of this semi-Delphi study we can conclude that the nature of the variable(s) of interest, reliability, feasibility and statistics were important to keep in mind when making decisions on diary design topics. Small differences in importance scores were found. The nature of the variable(s) of interest (e.g., its occurrence or fluctuation rate) was found to be most important for making a decision about the measurement frequency and the choice for momentary or retrospective assessments. The nature of the variable(s) of interest, and the reliability (e.g. obtaining representative data) were found to be most important for the choice of the study duration. The reliability and statistics (e.g. obtaining equidistant data) were most important for the choice of (semi)random or fixed assessment. The nature of the variable, reliability and feasibility were all found equally important for the choice of the delay allowed to respond.

The strong points of this study are that the qualitative approach allowed insight into the reasoning of researchers when deciding on a particular study design. Moreover, researchers from eleven different countries from 27 institutes participated and reported about a wide variety of studies which increased the generalizability of our study. The open text field answers were independently scored by two reviewers, in order to diminish subjective choices while grouping the reported reasons.

A limitation of this study is that although we planned to perform a Delphi study, the diversity and the number of topics addressed did not allow in depth discussion of different viewpoints of participating researchers. For example, most researchers argued that increasing the measurement frequency would increase participant burden, while some reported that increasing the measurement frequency actually decreases participant burden, since it becomes more routine for participants to fill out the questionnaire. It would have been interesting to have the participating researchers discussing these different points of view, for example in focus groups. Moreover, the online survey might potentially have led to less extensive responses than could have been obtained by face-to-face interviews. Further, although we strived to include researchers from a variety of research fields by inviting researchers who were member of the Society of Ambulatory Assessment, we only partially managed to do so. By also contacting researchers from our personal network, an oversampling might have occurred of researchers using fixed and low frequency sampling schemes and of researchers performing studies on mood disorders. Also some participating researchers were relatively new in the field, and completed only one or two diary studies so far. This might have diminished the generalizability of our findings.

Findings in the current study are generally in line with recommendations in text books and other methodological articles in which diary design topics are addressed [1, 4–6, 28], and no unexpected findings came out. The emphasis on particular diary designs in prior publications was however somewhat different from the current study, as they were mostly in favour of (semi)random designs and momentary assessment. The current study found also many arguments in favour of using fixed designs and retrospective assessment. This is probably due to the larger weight that was given to statistical possibilities for data collected at equidistant time points and the nature of the variable of interest for choices of diary designs. In the current study, also topics that so far gained relatively little attention were addressed such as the number of items to include in a diary study and the time participants were allowed to respond to the prompt. Further, the current study is the first to identify and categorize reasons for diary design choices made by researchers from different research fields in specific diary studies. Therefore, it offers examples of translations of methodological knowledge to specific research settings. Upcoming researchers in the field might thereby obtain further insight into the various options and to consider these options carefully when planning a study. Researchers are made aware that these choices may have a large influence on the collected data and on the research questions that can be answered. We therefore believe that our study might serve as a helpful source of information for researchers designing diary-based research. It presents an overview of the different choices they can make, with arguments in favour of specific choices in specific circumstances. It also shows that design choices often are a trade-off between different themes, because taking the nature of the variables, reliability, feasibility, and statistical possibilities into account when choosing a specific design topic may lead to conflicting optimal designs. For example, a long study duration may improve reliability, but decrease feasibility. Most importantly, we hope to increase the awareness that a gold standard for the optimal design of a diary study is not possible, since the design depends heavily on the research question.

To make the results more applicable for future researchers, we developed a checklist for designing a diary study based on our results. This checklist is intended to make researchers think carefully about their research design before conducting a diary study, and contains practical considerations, such as sending out reminder text messages. The checklist is given in Additional file 3 and is successfully used at our department. Results of our study can also be used to adapt the recently published checklist for reporting EMA studies that was based on the Strengthening the Reporting of Observational Studies in Epidemiology (STROBE) checklist [9]. For example, information on reasons behind design selection decisions, information on whether items were assessed momentary or retrospectively, and information on the delay the respondents were allowed to respond might be useful additions to this checklist, since this information might help other researchers when designing their own study. Further, information on whether items were assessed momentarily or retrospectively is essential for interpreting the results. Information on the dynamics of the variables of interests and the participant burden of different sampling schemes is also essential for making sophisticated decisions. These topics have up-till-now only been scarcely examined (e.g., [2, 27, 29]). We believe that a careful description of diary designs in the method section of future studies or on study pre-registration platforms might increase insight into these topics.

## Conclusions

The current study identified different topics that are helpful to keep in mind when designing a diary study, namely the nature of the variables, reliability, feasibility and statistics. All these topics were found to be important for choices on the study duration, the measurement frequency, random or fixed assessment, momentary or retrospective assessment, and time allowed to respond to the beep. No preferred designs have been provided, since the exact choices for the study design depend heavily upon the research questions. We believe this study will help guiding the choices that have to be made for optimal diary designs.

## Additional files

Additional file 1: Additional practical suggestions as reported by participating researchers. (DOCX 16 kb)

Additional file 2: Additional gaps in the literature as reported by participating researchers. (DOCX 15 kb)

Additional file 3: Example of a checklist for handing in a diary study used within our (i.e. the authors) psychiatry department at the University Medical Center Groningen, the Netherlands. (DOCX 18 kb)

## Abbreviations

EMA: Ecologically momentary assessment
ESM: Experience samping methods
IQR: Inter quartile range
