# Supplementary material for: A qualitative approach to guide choices for designing a diary study
Source: BMC Med Res Methodol. 2018 Nov 16;18:140. doi: 10.1186/s12874-018-0579-6 (PMC6240196; doi:10.1186/s12874-018-0579-6)
Supplement: Supplementary file 1 — Additional practical suggestions as reported by participating researchers. (DOCX 16 kb) [file 12874_2018_579_MOESM1_ESM.docx]

**Additional file 1. Practical suggestions as reported by participating researchers**

| **Reliability**   - Align with timeline follow-back timeframes [note by the authors: timeline follow-back timeframes is a method that can be used as a clinical and research tool to obtain a variety of quantitative estimates of marijuana, cigarette, and other drug use] - Predefined rules over what data (e.g. with regard to delay) to accept for statistical analyses - Assess at least three items for each construct you aim at - Realize that the reliability differs per variable (depends on its nature)   **Feasibility**   - Adding an extra assessment in case of technical problems - Questionnaire disappears when next pops-up - Questionnaire appears when respondent plugs in his/her telephone for charging - Not all items have to be asked equally frequent - Reduce branching   **Statistics**   - Use simulation studies - Contact a statistician before conducting your study - Use an e-diary that is triggered in response to an interesting episode of one continuously measured variable - Aim for consistent answering scales (momentary/retrospective or likert-scale/ continuous) - Use a combination of momentary and retrospective items |
| --- |

*Please note that participating researchers were sometimes short and unspecific in their answers*
