# Supplementary material for: A qualitative approach to guide choices for designing a diary study
Source: BMC Med Res Methodol. 2018 Nov 16;18:140. doi: 10.1186/s12874-018-0579-6 (PMC6240196; doi:10.1186/s12874-018-0579-6)
Supplement: Supplementary file 2 — Additional gaps in the literature as reported by participating researchers. (DOCX 15 kb) [file 12874_2018_579_MOESM2_ESM.docx]

**Additional file 2. Gaps in the literature reported by participating researchers**

| - Whether dynamics of the variables differ for specific groups - Information on remuneration effects - Guidelines for compensation of participants - Information on what is more important: equidistance in chronological time or distance in lived time (e.g. time between getting up and heaving lunch) - Impact of the gap in time-series data due to the night on statistical results - Whether observations of people who miss many data entries are less reliable - Recall bias of different constructs - Test-retest reliability of diary studies - Whether participating in a diary study influences the answers - How much delay to respond to the beep to allow - Apps to assess alcohol or drugs intake - Standardized ESM items - Information on how to design a good ESM item |
| --- |

*Please note that participating researchers were sometimes short and unspecific in their answers*
