# Supplementary material for: A qualitative approach to guide choices for designing a diary study
Source: BMC Med Res Methodol. 2018 Nov 16;18:140. doi: 10.1186/s12874-018-0579-6 (PMC6240196; doi:10.1186/s12874-018-0579-6)
Supplement: Supplementary file 3 — Example of a checklist for handing in a diary study used within our (i.e. the authors) psychiatry department at the University Medical Center Groningen, the Netherlands. (DOCX 18 kb) [file 12874_2018_579_MOESM3_ESM.docx]

**Additional file 3. Example of a checklist for handing in a diary study used within our department**

*Please note that this checklist contains information that is specific for our department, within our department RoQua is an IT support service for performance of diary studies (www. roqua.nl). for this manuscript references to specific places are pointed to in Italics*

Sampling protocol

- What is the study duration? *For more information on choosing the study duration, please see paragraph Results- Study duration*
- Do you want the diary to stop automatically or do you stop the diary protocol yourself?
- What is the sampling frequency? *For more information on the sampling frequency, please see paragraph Results- Measurement frequency*
  - If only once a day, at what times of the day participants have to fill out their diaries?
  - If the sampling frequency is more frequently than once a day, what is the interval between the assessments? *Please note that you can choose the exact time of the first diary assessment yourself for each participant, these time-points are fixed per participant during the entire assessment period and random assessment is not yet possible within RoQua.*
- What is the delay allowed to respond to the beep? *For more information, see paragraph Results- Allowed delay to respond*
- Do you wish to send text-message (SMS) notifications to prompt participants to fill out their diary; how many and when?
- Do you wish to send SMS reminders to remind participants that they did not fill out their diary; how many and how many minutes after the notification?
- Do you wish to send e-mail notifications; when?
- Do you wish to send e-mail reminders; how many and when?
- Do you want to include one trial/ instruction assessment in which you guide the participant with filling out the diary at the beginning of your study?
- Do you want to include a trial period, during which the participant can get familiar with filling out the diary? How long should this period take?

Content

- Provide the items of your diary questionnaire in the questionnaire in the exact wording you want to have them. *For more information on questionnaire development, see (1)*
- Did you think carefully about this number of items? *For more information see Table 2 and paragraph Results- Study Intensity*
- Did you think carefully about whether you want to assess items momentary or retrospectively? *For more information see paragraph Results- Retrospective or momentary assessment*
- Which response format should be used for your questions (e.g. Likert scale, multiple option checkbox, open text area, slider)
  - If a slider scale is used, should a selection box been shown immediately or appear after a participant clicked on the scale?
  - If a Likert scale is used, please be aware that a neutral position is only possible with unequal scales (e.g. 7 point or 11 point)
  - Which labels do you want for your scales?
  - Where do you want the labels: only at the end points of the scales or also in the middle?
- Do you want to personalize the diary items that are assessed? If yes, which items?
- Do you have an item that is designed by the participant*?* If yes, please provide.
- Do you want to use branching? If yes, which items? *Please note that this is only possible in RoQua based on answer on a dichotomous or Likert-scale, and not possible based on a slider scale or open text field.*
- Which questions are obligatory?
- Do you want all questions to appear on one page or to divide your diary into separate sections?
- Do you want to include a morning diary to make it possible to ask some questions only once a day (e.g. about sleep)?
- Do you want to include an evening diary to make it possible to ask some questions only once a day (e.g. about alcohol use)?

Communication with participants, please hand in:

- A mail template for your trial/instruction session [only if you want a trial/instruction session]
- A SMS template (max 160 characters) for inviting your participants to fill out the diary
- A SMS template for the SMS reminder [only if you do wish to use a SMS reminder]
- Mail template for inviting the participant to fill out the diaries, eventually more than one if you have different diaries (e.g. morning/evening) *We do recommend SMS reminders instead of mail reminders if you measure > 1 a day.*
- A single send mail template containing the link for filling out the questionnaires, the participants could save this mail to easily access the questionnaires
- A mail template for your reminder mail [only if you wish to use a reminder mail

Use of extra options

- Do you want to include a feasibility questionnaire to evaluate your study? If yes, hand in the items for this questionnaire as well.
- Do you want RoQua to develop an automatic feedback report for each participant based on the diary data? If yes, please contact RoQua to discuss the details.

Organizational aspects

- Are you aware of the total costs for performing your diary study within RoQua (e.g. SMS costs, development of diary protocol, development of feedback report [optional])?
- Are you aware that you have to perform a test run (with non-patient participants) to test your diary before you can start conducting your study?
- Are you aware that it can take several weeks before you can start conducting your study once you have handed in your diary protocol?
